# Peripheral T-cell lymphoma, NOS in bone marrow and heart

**DOI:** 10.1007/s44313-024-00009-7

**Published:** 2024-02-26

**Authors:** Hye Won Lee, Ja Young Lee

**Affiliations:** Department of Laboratory Medicine, Busan Paik Hospital, Inje University College of Medicine, 75 Bokji-Ro Busanjin-Gu, Busan, 47392 Korea

**Keywords:** Lymphoma, T-Cell, Peripheral, Flow cytometry, Bone marrow, Heart

A 65-year-old man was admitted presenting dyspnea aggravated by changes in posture. Three months earlier, he had been diagnosed with tuberculosis pericarditis and hypertension. Peripheral blood smear revealed the presence of atypical large lymphocytes characterized by azurophilic cytoplasmic granules (1%) and severe thrombocytopenia (platelet count of 40 × 10^9^/L) (A, Wright-Giemsa stain, 1,000 ×). Bone marrow (BM) aspiration revealed an increased proportion of large lymphocytes, reaching up to 56.4% (B, Wright-Giemsa stain, 1,000 ×). Flow cytometry analysis identified an expanded CD4 + /CD8 + mature T-cell population with aberrant expression of CD19 and CD56, and a predominant CD8 expression (C, T-lymphoid cells appear red except in the last figure, in which CD8-dominant lymphoid cells appear blue). Chromosomal analysis of BM aspirate showed malignant clones exhibiting a complex karyotype. A subsequent pericardial biopsy confirmed the diagnosis of peripheral T-cell lymphoma with cardiac and BM involvement. Chemotherapy was initiated with cyclophosphamide, doxorubicin, vincristine, and prednisolone. Unfortunately, the patient died two weeks after diagnosis. Cardiac T-cell lymphoma with BM involvement is extremely rare. Due to the aggressive and rapidly expanding nature of primary cardiac lymphoma—a life-threatening malignancy—prompt and precise diagnosis and treatment are crucial.
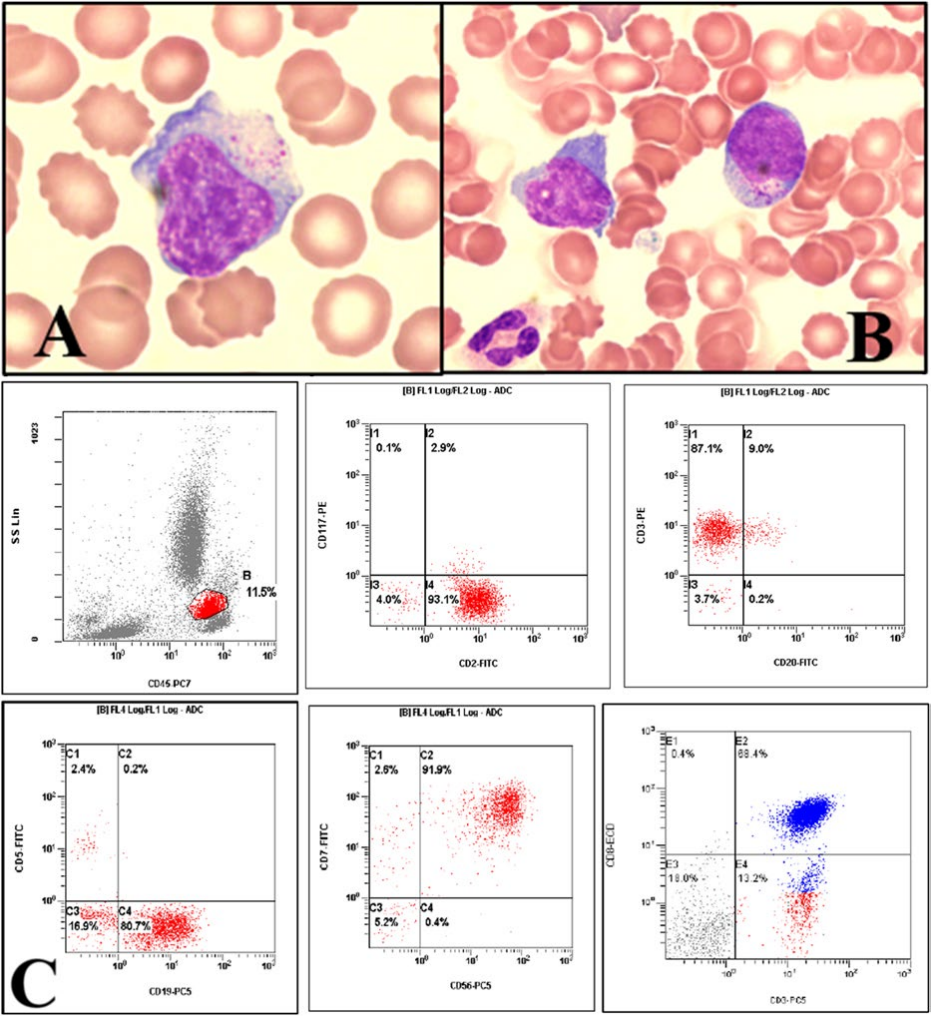


## Data Availability

No datasets were generated or analysed during the current study.

